# Same day ART initiation versus clinic-based pre-ART assessment and counselling for individuals newly tested HIV-positive during community-based HIV testing in rural Lesotho – a randomized controlled trial (CASCADE trial)

**DOI:** 10.1186/s12889-016-2972-6

**Published:** 2016-04-14

**Authors:** Niklaus Daniel Labhardt, Isaac Ringera, Thabo Ishmael Lejone, Phofu Masethothi, T’sepang Thaanyane, Mashaete Kamele, Ravi Shankar Gupta, Kyaw Thin, Bernard Cerutti, Thomas Klimkait, Christiane Fritz, Tracy Renée Glass

**Affiliations:** Clinical Research Unit, Medical Services and Diagnostics, Swiss Tropical and Public Health Institute, Socinstrasse 57, 4051 Basel, Switzerland; University of Basel, Basel, Switzerland; SolidarMed, Swiss Organization for Health in Africa, Premium House #224, Kingsway, P.O.Box 0254, Maseru West, 105 Lesotho; District Health Management Team Butha-Buthe, Ministry of Health of Lesotho, Butha-Buthe, Lesotho; Research Coordination Unit, Room Number 326, Ministry of Health of Lesotho, Maseru, Lesotho; Faculty of Medicine, UDREM, University of Geneva, 1 Rue Michel Servet, 1211 Geneva, Switzerland; Department of Biomedicine – Petersplatz, Molecular Virology, University of Basel, Basel, Switzerland; Biostatistics Department, Epidemiology and Public Health Unit, Swiss Tropical and Public Health Institute, Socinstrasse 57, P.O. Box 4002, Basel, Switzerland

**Keywords:** Linkage to care, Retention in care, Viral suppression, HIV, Antiretroviral therapy, Lesotho, Africa, Randomized controlled trial, Care cascade

## Abstract

**Background:**

Achievement of the UNAIDS 90-90-90 targets in Sub-Sahara Africa is challenged by a weak care-cascade with poor linkage to care and retention in care. Community-based HIV testing and counselling (HTC) is widely used in African countries. However, rates of linkage to care and initiation of antiretroviral therapy (ART) in individuals who tested HIV-positive are often very low. A frequently cited reason for non-linkage to care is the time-consuming pre-ART assessment often requiring several clinic visits before ART-initiation.

**Methods:**

This two-armed open-label randomized controlled trial compares in individuals tested HIV-positive during community-based HTC the proposition of same-day community-based ART-initiation to the standard of care pre-ART assessment at the clinic. Home-based HTC campaigns will be conducted in catchment areas of six clinics in rural Lesotho. Households where at least one individual tested HIV positive will be randomized. In the standard of care group individuals receive post-test counselling and referral to the nearest clinic for pre-ART assessment and counselling. Once they have started ART the follow-up schedule foresees monthly clinic visits. Individuals randomized to the intervention group receive on the spot point-of-care pre-ART assessment and adherence counselling with the proposition to start ART that same day. Once they have started ART, follow-up clinic visits will be less frequent. First primary outcome is linkage to care (individual presents at the clinic at least once within 3 months after the HIV test). The second primary outcome is viral suppression 12 months after enrolment in the study. We plan to enrol a minimum of 260 households with 1:1 allocation and parallel assignment into both arms.

**Discussion:**

This trial will show if in individuals tested HIV-positive during community-based HTC campaigns the proposition of same-day ART initiation in the community, combined with less frequent follow-up visits at the clinic could be a pragmatic approach to improve the care cascade in similar settings.

**Trial registration:**

NCT02692027, registered February 21, 2016

## Background

In November 2014 the Joint United Nations Programme on HIV/AIDS (UNAIDS) published the 90-90-90 targets for 2020 [1]. The strategy aims at a massive scale-up in coverage of antiretroviral therapy (ART) among individuals infected with HIV. Based on accumulated evidence that viral suppression through successful ART reduces the risk of transmission [2, 3], it is expected that – if achieved – the 90-90-90 targets would lead to a reduction of the yearly global HIV-incidence from 2 million currently to 500,000 by 2020 [4]. In 2015 two randomized controlled trials showed the benefit of starting ART as early as possible for infected individuals – even if CD4-cell counts were above the threshold of 500 cells/mL [5, 6], leading the World Health Organization (WHO) to recommend that anyone infected with HIV should start ART as soon as possible after diagnosis [7]. A “seek-test-treat” strategy bears, however, unprecedented challenges in settings where HIV is hyperendemic and resources may be limited [8, 9].

The Continuum of Care Cascade (“the cascade”) involves the steps HIV-infected individuals have to take in order to achieve viral suppression. It starts with knowing one’s HIV status, continues with linkage to care after a positive HIV test, initiation of ART, uninterrupted continuation of ART (retention in care and adherence to medication), and ends with sustained viral suppression [10]. Already prior to announcement of the “seek-test-and-treat” approach, weaknesses in the cascade often hampered the effectiveness of HIV programs in resource-rich as well as resource-poor settings [11–13]. In Sub-Saharan Africa the care cascade is still far from the 90-90-90 targets with only 29 % of infected individuals estimated to be on ART and virally suppressed in 2013 [1]. In order to achieve the UNAIDS targets innovative, effective, and practical approaches for improving the care cascade are thus urgently needed [14, 15].

Linkage to care after an initial positive HIV test has been described as the “Achilles’ heel” of the care cascade [16]. Most studies from Sub-Saharan Africa report linkage rates lower than 50 % [17–21]. In a cluster-randomized trial comparing home-based with mobile-clinic HIV testing and counselling (HTC) in Lesotho, only 25 % of newly tested HIV-positive individuals accessed care within 1 month after the test [22].

Several interventions have been shown to improve linkage to care, such as point-of-care CD4 count directly after a positive HIV test [23], immediate start of cotrimoxazole prophylaxis [24], incentives such as food-assistance [25], extended post-test counselling during home-visits, or community-workers accompaniment [26–28]. However, controlled studies testing programmatic intervention packages for improving linkage to care are still largely lacking [29, 30]. Furthermore, it must be noted that interventions, such as patient-accompaniment or food support are resource intensive and may work in small NGO-driven projects, but are not sustainable on a larger scale [31]. In a systematic review addressing barriers for linkage to care, transport cost and distance were the most frequently cited factors for patients not enrolling in care after a positive HIV test [32].

This paper describes the protocol of the CASCADE-trial. This trial tests the effectiveness of same day home-based ART initiation after a positive HIV test in combination with a reduction of the frequency of follow-up visits to the clinic as a pragmatic and programmatically feasible approach to improve linkage to care, retention in care, and viral suppression.

## Methods

### Trial design

The CASCADE-trial is a two-armed open-label randomized controlled trial. Allocation is 1:1 with parallel assignment. The intervention is targeted to individuals who tested HIV-positive during community-based HTC and entails same day community-based ART start combined with less frequent follow-up visits. The intervention arm will be compared to the standard of care in Lesotho (referral to facility for ART initiation followed by monthly follow-up visits). The primary outcomes are linkage to care at 3 months and viral suppression at 12 months after a positive HIV test.

### Recruitment and participants

Participants will be recruited during community-based HTC campaigns in the district of Butha-Buthe, in northern Lesotho. Lesotho has an estimated adult HIV-prevalence of 23.4 %. Among the hyper-endemic countries of southern Africa it has the highest transmission rate and the lowest ART coverage [33]. Butha-Buthe is a rural district with an estimated 110,000 habitants, mostly subsidence farmers or mine workers, or construction or domestic workers in neighboring South Africa. The district has only one small town (Buthe-Buthe, ca. 25,000 habitants), the remaining habitants live in villages scattered over a partly mountainous surface of 1,767 km^2^. According to UNAIDS an estimated 11,000 HIV-infected adult persons lived in the district in 2013 [34].

Home-based HTC campaigns will be conducted for an anticipated period of 3 months beginning at the end of February in the catchment areas of six health care facilities - four nurse-led health centers, one missionary and one public hospital.

For rural areas, villages were randomly selected from a list of eligible villages. A village is eligible if it is clearly confined to the catchment area of one of the six facilities and if it comprises between 40 and 80 households. In Butha-Buthe town urban neighbourhoods were randomly selected.

Three teams consisting of 4 lay counsellors, each supervised by one professional counsellor and a nurse visit all households in selected villages and urban neighbourhoods proposing HTC to all household members. Each house will be visited once during the workweek. During this first visit, lay counsellors enquire about absent household members. If a household member is absent, the household will be visited a second time during a weekend. The HTC-coverage in visited areas will be assessed as part of a nested study (see nested studies below).

If a household member tests HIV positive during the HTC campaign, the study nurse is called to assess whether the individual is eligible for the CASCADE trial. Consenting individuals 18 years or older, living or working in the district of Butha-Buthe, having never been on combination ART and willing to receive follow-up at one of the six study facilities are eligible. Exclusion criteria are: pregnant or breast-feeding, already enrolled in care for another chronic disease, clinical WHO stage 4 or active tuberculosis, or a positive cryptococcal antigen test.

If the sample size will not be reached during the 3 months of home-based HTC campaigns, the recruitment period will be extended and mobile clinics will be set up at urban or rural spots, such as taxi ranks or market places. Procedures for home-based HTC as well as HTC in mobile clinics in Lesotho has been described in detail elsewhere [22].

### Control and intervention arms

The control group follows the standard of care in Lesotho [35, 36] that is similar to most settings in Sub Saharan Africa. During the household visit, individuals found HIV-positive and randomized to the control arm will receive post-test counselling and a referral letter with an appointment at their chosen health facility. On the first visit to the clinic, the participant receives a laboratory assessment (CD4 cell-count, serum creatinine, haemoglobin) and a first adherence counselling session. The participant must then return to the clinic to receive his/her laboratory results and to undergo a second adherence counselling session. Depending on whether the participant is judged as “ready to start ART” by the counsellor at the clinic, he/she may start ART at their second clinic visit or have to attend a third adherence counselling session. Once the participant has started ART, the first and second follow-up visits are scheduled for 14 and 28 days after ART initiation, respectively. Thereafter, follow-up visits are scheduled monthly until 6 months after ART initiation. If the participant is clinically stable, clinical follow-up visits may then be spaced to 3-monthly intervals, but refills of ART must still be collected on a monthly basis.

Participants randomized to the intervention group receive post-test counselling and clinical and laboratory assessment with the possibility of same day ART start combined with a reduced frequency of follow-up visits. Directly after the positive HIV-test result the study nurse assesses the participant clinically and performs point-of-care laboratory testing (serum creatinine (StatSensor Creat®, Nova® Biomedical), haemoglobin (Hemocue®, HB301), and CD4 cell-count (PIMA Alere®)). Thereafter, the participant receives a standardized short adherence counselling. After adherence counselling, if – according to the nurse’s judgement - the person understood the implications of lifelong ART, he/she will be offered to start ART immediately. Those who decide to start ART during the visit or intend to start within 7 days will receive a 30-day ART-supply and a follow-up appointment at the clinic within 12 to 16 days. Choice of ART regimen and use of cotrimoxazole prophylaxis follows national and international guidelines [36, 37]. Participants with a CD4 cell-count < 100 cells/mL will receive testing for cryptococcal antigen. In case of a positive result, the participant will be treated according to guidelines and excluded from the study. In order to reduce travel time and transport cost, participants who are clinically stable, will have their follow-up visits and ART refills more widely spaced with appointments at 6 weeks, 3, 6, (9) and 12 months after ART initiation. Participants are, however, encouraged to visit the clinic at any time in case of problems or questions.

**Fig. 1:**
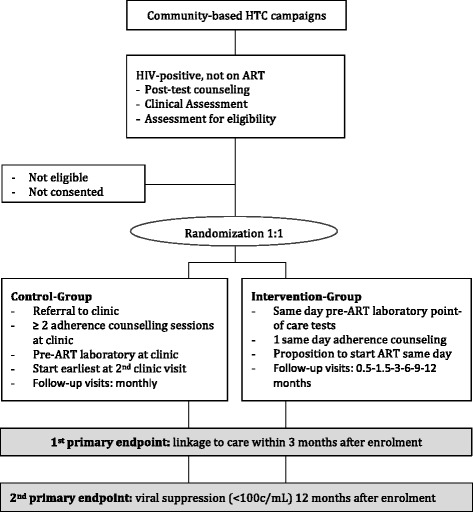
CONSORT flow-chart of the CASCADE-trial. HTC: HIV testing and counselling; ART: anti-retroviral therapy

Figure 1 summarizes flow of participants in the study.

**Table:** 

Service provided	Control group	Intervention group
Baseline laboratory values (creatinine, CD4-count, haemoglobin)	Blood taken at first clinic visit after the positive HIV test result, results communicated at second clinic visit.	Performed in the community the day of the HIV test result as point-of-care
Adherence-counselling	At first and second clinic visit	In the community, directly after the HIV test result
ART-initiation	After at least two adherence counselling sessions at the clinic	Proposition of starting the same day as tested HIV positive
Follow-up visits and drug-refills at the clinic	Monthly	2 weeks – 6 weeks – 3–6–9–12 months

Table 1 Overview of services provided after a positive HIV test result during community-based HTC campaigns in the control and intervention group

### Primary outcomes

The trial has two primary outcomes. The first primary outcome is linkage to care within 3 months after having been tested HIV positive during the HTC-campaign. A patient is considered to have linked to care if he/she attends the clinic at least once within 90 days after enrolment in the trial. The second primary outcome is viral suppression 12 months after enrolment in the trial. Viral suppression is defined as a viral load (VL) <100 copies/mL between 11 and 14 months after diagnosis of HIV-infection.

### Secondary outcomes

Secondary outcomes are defined in Table 1.

**Table 1:** Secondary outcomes

Secondary outcome	Definition	Time-frame	Remarks
ART-initiation	Participant started ART	3 months after the enrolment	
1-year retention in care	Proportion confirmed dead (death record at clinic or confirmed by a first-grade relative), lost to follow-up (not attending the clinic 11–14 months after HIV-test and not confirmed dead), and retained in care (on ART, attends the clinic)	12 months (11–14 months) after enrolment	Participants with transfer out to another clinic are excluded from analysis. Participants are considered as transferred out if the facility where they were enrolled in care issued a transfer letter.
Viral suppression under ART	VL<100 copies/mL among those who started ART	6 months (5–7) months) after ART initiation	
Change in body weight	Delta between body weight (kg) at baseline and 12 months after enrolment	At baseline and 12 months (11–14 months) after enrolment	
Change in CD4 cell count	Delta between CD4 cell count (cells/mL) at baseline and 12 months after enrolment	At baseline and 12 months (11–14 months) after enrolment	
Change in haemoglobin	Delta haemoglobin (g/dL) at baseline and 12 months after enrolment	At baseline and 12 months (11–14 months) after enrolment	
New clinical WHO stage 3 or 4 events	Diagnosis of a new clinical WHO stage 3 or 4 event confirmed by study-physician	12 months (11–14 months) after enrolment in the trial	WHO 3 or 4 events occurring within 3 months after ART-initiation and judged as immune reconstitution inflammatory syndrome by the study physician will not be classified as a new WHO stage 3 or 4 event

### Sample size and randomization

Households will be randomly allocated to a study arm at a ratio of 1:1. A randomization list was generated by an independent statistician with a block size of 4. Sealed and consecutively numbered envelopes containing treatment assignment and study documents are provided to the study nurses. It was not possible for treatment to be blinded.

The sample size for this trial is based on the primary endpoint of linkage to care within 3 months after HIV diagnosis. We expect 3-month linkage to care rates of 40 % in the control and 60 % in the intervention arm. As the trial is randomized at the household level, we require a total of 260 households with at least one HIV-positive participant to detect a 20 % increase in linkage to care assuming a Type I error rate of 5 % and power of 90 %. With an estimated uptake of HTC of 94 %, a prevalence of previously untreated HIV infection during home-based HTC campaigns of 5 %, and a 10 % trial participation refusal rate or choice of alternative health facility, we need to visit 6200 households to achieve the desired sample size of 260 households. In case more than one HIV-positive individual is diagnosed in the household, all individuals meeting eligibility criteria will be included and randomized to the same arm. This will only serve to increase the power of the study.

With the proposed sample size above, we will have sufficient power to test the second primary endpoint of viral suppression at 12 months. As those not linking to care will be considered as failures, we expect viral suppression rates of 25 % in the control arm and 45 % in the intervention arm. With 130 individuals per arm, we will have 93 % power to detect a difference of 20 % in viral suppression rates.

Analysis will be as intention to treat. The study will be analysed using mixed effects logistic regression models in order to account for the clustering of households (if sufficient number of households include more than one HIV-positive individual in the study) and village. If the arms are not found to be balanced on important risk factors for linkage to care and viral suppression, these risk factors will be adjusted for in models. Linkage and viral suppression rates between the control and intervention arm will be presented with odds ratios and 95 % confidence intervals.

### Subgroup analyses

Individuals working outside of Lesotho (primarily in South Africa) are considered as a special subgroup as they usually spend several months out of the country at a time. This makes them an especially challenging group to link to care, but once linked, they are typically given refills and appointments every 3 months to accommodate their work schedule. Rather than exclude these individuals, we assume they will be evenly distributed among the treatment arms and they will be analysed in a separate subgroup analysis. As this group has rarely been studied, we do not have valid estimates of their expected linkage to care or viral suppression rates. In addition, we do not expect to have the power to sufficiently test for differences among these groups but nevertheless find their results of great interest for the design of future interventions targeting this difficult-to-treat population.

### Data collection and management

During the HTC-campaigns that serve as recruitment for the CASCADE trial, lay counsellors, under the supervision of a professional counsellor, will capture data on households visited and uptake and results of HTC using a tablet-based app and platform (Visible Solutions AG, Switzerland, visibleimpact.org). Data are stored in banking-standard ISO 27001 audited data centers. Aggregated data on number of households visited and number of individuals tested are regularly updated and publicly available on https://visibleimpact.org/projects/1197-cascade-trial.

For individuals testing HIV-positive and participating in the CASCADE-trial further documentation will be done by study nurses on standardized paper-based forms. These forms will be scanned in Lesotho and subsequently processed with Data-Scan 5.7.7 (Neoptec, Montpellier, France) for electronic data capture.

### Storage of plasma

At recruitment and after 12 months of follow-up, plasma samples of the trial participants will be stored at -80° at the laboratory in Butha-Buthe hospital to allow retrospective resistance testing in case of treatment failure.

### Ethics

The trial protocol has been approved by the Ethical Board in Switzerland (Ethikkommission Nordwestschweiz; EKNZ UBE 15/123) and the National Health Research and Ethics Committee of the Ministry of Health of Lesotho (89-2015). For the home-based HTC campaigns the head of household or his/her substitute must provide written informed consent to the lay counsellor to visit the household and to propose HTC to household members who are present. As per Lesotho National HTC guidelines each individual who agrees to HIV testing must sign the National HTC Consent Form. In case an individual is eligible for the CASCADE-trial he or she will be asked for written informed consent. Individuals who are not able to sign with their name can provide a thump-print.

Individuals who participate in nested study 2 as HIV-negative controls sign a specific informed consent form for blood draw and analysis of their lipid-status and glycosylated haemoglobin (HbA1c) (see below for nested study 2).

Individual participating in the CASCADE trial or one of the nested studies will not receive any financial compensation.

### Trial status

Recruitment started with launch of the home-based HTC campaigns on February 22, 2016. The status of recruitment can be followed on https://visibleimpact.org/projects/1197-cascade-trial/show/about. Recruitment is anticipated to finish by the end of May 2016. The recruitment phase may, however, be extended if the targeted sample size is not achieved. The trial is expected to be completed in September 2017.

### Nested studies

The trial hosts two nested studies. Nested study 1 is a cross-sectional study assessing the testing coverage that is achieved with two home-based HTC visits to the randomly chosen areas for HTC-campaigns during recruitment for the CASCADE-trial. Based on the number of individuals tested and the total number of persons living in the visited households of the testing areas, the HTC-coverage after the first and second visit will be calculated. This study will provide information on feasibility and resources needed to achieve the targeted 90 % HTC coverage through home-based HTC when each area is visited twice, once during the work week and once on a weekend day, as described above.

Nested study 2 assesses lipid-profiles (total cholesterol, LDL, HDL cholesterol, triglycerides) and glycosylated haemoglobin levels (HbA1c) among ART-naïve HIV-infected individuals compared to non HIV-infected controls. For the cohort of HIV-infected individuals who initiate ART within the CASCADE-trial lipid-profiles and HbA1c levels will again be assessed six and 12 months after ART-initiation.

All eligible and consenting HIV-positive individuals in the CASCADE trial (*n* = 260) will be included in the sub-study. A target of 260 HIV negative controls will be recruited from the same household as the HIV-positive individuals. Only one HIV-negative individual will be selected from each household with a preference for the household member most closely matching the HIV-positive individual in terms of gender and age. In case there is no eligible HIV-negative individual in the same household, the next eligible individual from the nearest household will be enrolled.

## Discussion

Community-based HTC campaigns will be one important intervention towards reaching the 90-90-90 goals as it is a key strategy to access “hard-to-reach” populations [38]. Despite recent popularity of home-based HTC campaigns, linkage after a positive HIV test continues to be uniformly poor in Sub Saharan Africa [16, 39] and interventions improving linkage to and subsequent retention in care are greatly needed [40]. The combination of interventions tested in the CASCADE-study could represent a feasible add-on to the current practice of health-care-provider teams providing home-based HTC in rural and urban areas. If proven to be effective, this strategy will have the potential to be scaled up in similar settings without large amounts of additional resources. A recently conducted trial at a clinic in Johannesburg showed that clinic-based fast-track initiation of ART results in better linkage to care and higher viral suppression rates after a positive HIV test result at a clinic [41]. To our knowledge there are no trials assessing community-based same-day ART-initiation.

The CASCADE-trial will be among the first randomized controlled trials assessing community-based strategies to reach the 90-90-90 targets in a resource-limited HIV hyper-endemic setting. The trial has, however, several limitations. It includes only adult individuals who are tested during community-based HTC campaigns, thus excluding individuals testing at facilities as well as children. Moreover, after enrolment, participants may decide to access treatment outside the district of Butha-Buthe. This may lead to an underestimation of linkage to and retention in care in both arms.

According to UNAIDS in 2013 only 45 % of individuals were aware of their HIV-status, 39 % of diagnosed individuals received ART and 29 % of infected individuals had achieved viral suppression [1]. Implementation studies testing feasible and pragmatic approaches for improving the treatment cascade are urgently needed [14, 15]. The CASCADE-trial, will add evidence for future implementation guidelines and policies in Lesotho and similar hyper-endemic resource limited settings.

## Abbreviations

ART: antiretroviral therapy
HIV: Human Immune deficiency Virus
HTC: HIV Testing and Counselling
UNAIDS: Joint United Nations Programme on HIV/AIDS
VL: viral load
WHO: World Health Organization
